# Assessing Cultural Competence and Cultural Responsiveness of Healthcare Services That Promote Early Prevention of Childhood Obesity: A Scoping Review

**DOI:** 10.1111/obr.13979

**Published:** 2025-07-08

**Authors:** Zoe Chen, Sarah El Wazni, Erin Kerr, Huilan Xu, Li Ming Wen, Sarah Taki

**Affiliations:** ^1^ School of Medicine University of Sydney Sydney New South Wales Australia; ^2^ Health Promotion Unit, Population Health Research and Evaluation Hub Sydney Local Health District Sydney New South Wales Australia; ^3^ Sydney School of Public Health The University of Sydney Sydney New South Wales Australia; ^4^ The National Health and Medical Research Council Centre for Research Excellence in the Early Prevention of Obesity in Childhood (EPOCH CRE) Sydney New South Wales Australia; ^5^ Sydney Institute for Women, Children and Their Families Sydney Local Health District Sydney New South Wales Australia

**Keywords:** childhood obesity, cultural competence, culturally and linguistically diverse

## Abstract

**Background:**

Early childhood is a critical period to prevent future poor health outcomes. The modification of health behaviors in the first 2000 days of life is particularly crucial. Yet, obesity is increasingly prevalent in children from culturally and linguistically diverse (CALD) backgrounds, demonstrating a need to provide culturally‐tailored care to this population.

**Aims:**

To understand the extent of literature that explores cultural competence and responsiveness of preventive healthcare services delivered to culturally and linguistically diverse families (CALD) in the first 2000 days and to identify gaps in the literature and key characteristics and outcomes in these studies that are associated with culturally competent and responsive healthcare.

**Methods:**

A search strategy was developed, and five databases were searched. Title and abstract screening, full‐text screening, data extraction, and quality appraisal were performed by two or more independent reviewers.

**Results:**

A total of 28 included studies identified bilingual facilitators, language‐modified materials, and educational resources as key characteristics associated with culturally competent care. Improved breastfeeding practices and increased engagement with healthcare services were identified as key outcomes. Studies involving healthcare cultural competence training were associated with significant changes in outcomes.

**Conclusion:**

Many characteristics and health outcomes associated with culturally competent and responsive care were identified in this review. However, preventive health services delivered to CALD families are still an area of research and practice that is lacking cultural competence. A multidisciplinary and community‐centered approach is needed to improve health services delivered to CALD families and address persistent barriers to healthcare.

## 1 Introduction

Early childhood (i.e., 0–5 years old) is a critical time period to track child development and improve lifelong health outcomes. Studies have shown that children with increased adiposity at a younger age are at greater risk of developing future obesity and metabolic dysfunction, which includes insulin resistance, dyslipidemia, and hypertension [1]. These diseases represent the leading causes of death globally, as well as the greatest economic burdens to national health budgets [2, 3]. Research has shown that the in utero and early childhood experience play a key role in the onset of chronic diseases. During this period, risk factors such as maternal smoking during pregnancy, decreased length of breastfeeding, excessive gestation weight gain and newborn weight gain, family and environmental influences on diet, and decreased sleep duration all contribute to an increased risk of childhood obesity [2]. Other studies [1, 4, 5] highlight physical activity as an important preventive health target within early childhood. It has been demonstrated that establishing healthy behaviors during childhood to prevent chronic diseases is more effective and easier than attempting to reverse or change unhealthy behaviors in adulthood [5]. Obesity preventive health measures targeting diet, physical activity, sleep, and sedentary time have been shown to be the most effective when implemented in the first 2000 days of life, which marks conception up to 5 years of age [4, 5]. Policies, such as the First 2000 Days Framework [6] published in 2022, highlight the importance of instilling good health behaviors during childhood. Therefore, preventive health initiatives aimed at families in the first 2000 days targeting behaviors such as nutrition, sleep, and physical activity are paramount to addressing early childhood obesity.

Australia is home to a multicultural and diverse population with over 7 million overseas‐born Australians found in the 2021 census [7], most commonly born in England, India, China, New Zealand, and the Philippines. The most common languages spoken at home aside from English included Mandarin, Arabic, Vietnamese, Cantonese, and Punjabi. Many culturally and linguistically diverse (CALD) groups reside within Australia, where a person of CALD background is defined as someone who lives in an English‐speaking country but has come from a non‐English‐speaking country, has family from non‐English‐speaking countries, or speaks a range of languages [8]. Within Australia, 25% of children and adolescents were either overweight or obese between 2017 and 2018 [8]. A study investigating the risk of childhood obesity in children of CALD backgrounds in Victoria, Australia, reported that 53% of children from Middle Eastern backgrounds were obese or overweight compared to 36.7% of children from non‐CALD backgrounds [9]. There is a large disparity in obesity and other chronic disease prevalence in people from CALD backgrounds compared to their non‐CALD counterparts [9, 10, 11, 12]. This health disparity can be attributed to a number of factors, including different cultural and social norms towards dietary behaviors and physical activity, lowered health literacy rates, and decreased awareness of and access to healthcare services [13]. A study by Cyril et al. [14] identified further barriers to obesity preventive care engagement in CALD communities. The main barriers distinguished include competing lifestyle priorities, language and cultural barriers, lack of food and health literacy, exposure to junk food advertisements, and lack of compulsory childhood health checks. Systemic barriers such as provider bias, lack of cultural sensitivity in care, lack of clear policy guidelines on childhood obesity prevention, and inadequate services leading to long wait times also contribute to lack of access [15, 16, 17]. These studies all suggest that current preventive health measures are not as effective in certain populations, particularly CALD minority groups, contributing to health inequalities. There is an increasing need for preventive health services that cater to CALD groups.

Cultural competence in healthcare services and providers allows culturally sensitive and quality care to be delivered to CALD populations. Compounded by the growing number of CALD groups that populate member countries of the Organization for Economic Cooperation and Development (OECD) [18], which are contextually similar to Australia, cultural competence becomes especially crucial. Cultural competence is defined as a commitment to respecting and learning about other cultures and integrating cultural understanding into healthcare practices. Culturally‐tailored interventions delivered to target populations have been associated with better health outcomes [19]. Moreover, culturally‐competent healthcare plays a pivotal role in minimizing health disparities in healthcare access. Despite this, there is little research known in improving cultural competence of preventive healthcare services delivered to families from CALD backgrounds within the first 2000 days. As such, to appreciate the role of cultural competency in providing culturally appropriate and specific healthcare to improve the preventive health outcomes of children of CALD backgrounds, we must examine how cultural competency has been used in current literature in this context. This review aimed to identify gaps in the literature, as well as elucidate the key strategies, characteristics, and outcomes of studies that are effective in providing culturally‐competent care. The primary research question is: what is known from the literature on improving cultural competency of healthcare services and practitioners to support the preventive health of CALD families in the first 2000 days?

Sub‐questions are:

- What are the strategies and characteristics that are associated with culturally competent and responsive healthcare provided to CALD families in the first 2000 days?
- What are the preventive health outcomes associated with culturally competent and responsive care in these studies?

## 2 Methods

### 2.1 Protocol

The review protocol was guided by the JBI Methodology for Scoping Reviews [20] and published on Open Science Framework (https://doi.org/10.17605/OSF.IO/HA4M8).

### 2.2 Eligibility Criteria

#### 2.2.1 Inclusion Criteria

This review included peer‐reviewed original studies targeted at CALD families in the first 2000 days and conducted in OECD countries since 2013. Outcomes focused on the preventive health of children within the first 2000 days, including nutrition, physical activity, sleep, and oral health. Studies had to involve cultural competency—whether they were cultural adaptations of interventions, assessed cultural competence or cultural adaptations, involved health professional training, or evaluated patient or health professional experiences and perspectives. Included studies explored healthcare services provided by all medical practitioners, including general practitioners, dentists, or allied health professionals (e.g., nurses, physiotherapists and social workers).

#### 2.2.2 Exclusion Criteria

Studies were excluded if they were unpublished or ongoing, or any of the following: commentaries, review articles, opinion pieces, gray literature, or non‐human studies. Studies published outside of OECD nations, not in the English language, or translated into the English language, were excluded. Studies were excluded if they did not explore cultural competency in the following ways: assessments/interventions involving cultural competence, health professional cultural competence training, or patient experience. Studies involving translations only or adaptations of a measurement tool were excluded. Studies that targeted children over 5 years of age or adults, targeted non‐CALD participants, or targeted non‐preventive health measures and outcomes, and complementary medicine services or alternative health services were all excluded.

### 2.3 Information Sources

Databases used within this review included CINAHL via EBSCO, the Cochrane Database of Systematic Reviews, Embase via Ovid, Medline via Ovid, and Scopus. Databases were first searched in May 2023 and last searched in June 2023.

### 2.4 Search Strategy

Initial limited searches of Medline via Ovid and the Cochrane Database of Systematic Reviews were conducted to identify relevant articles. Consistent text words in titles and abstracts of identified articles were used to develop a search strategy for Medline via Ovid (see Appendix I). The search strategy was adapted to each database. Key search terms included (1) infants OR child* OR pre‐school, (2) AND cultur* AND (competenc* OR diversit* OR sensitivit*), and (3) AND (preventive health OR health promotion) OR diet OR physical activity OR sleep OR dental hygiene.

### 2.5 Selection of Sources of Evidence

Following the search, all identified citations were uploaded to Endnote 2020. Duplicates were removed, and citations were extracted and uploaded to Covidence. Titles and abstracts were screened by five independent reviewers (S.W., Z.C., L.W., E.K., and H.X.) for assessment according to the inclusion criteria. Following this, an evaluation of the full text was conducted by two independent reviewers (S.W. and Z.C.). Articles where full texts could not be found were excluded due to time constraints. All disagreements between screeners were resolved by discussion or an additional independent reviewer (S.T.).

### 2.6 Data Charting Process

#### 2.6.1 Data Extraction

Data were extracted by two independent reviewers (S.W. and Z.C.) following the extraction template in Appendix II adapted from the JBI Methodology for Scoping Reviews [20]. The template was drafted and piloted with five studies and further refined due to differences in study types and components of cultural competence. Data extracted included details about the study (e.g., title, author, publication year and origin), target population (e.g., description, setting and recruitment), methodology (e.g., study design and duration), and key outcomes and findings. The last section reported on the component of cultural competence, which included details of how cultural competence was explored, a description of the process, the rationale, the extent of involvement of the target group, and who led the cultural competence process. A summarized table of results can be found in Table 1.

**TABLE 1:** General characteristics of included studies (*n* = 28).

First author, year, reference, country	Aim of study	Study design	Target population and setting	Reported limitations
Asfour 2015 [21], United States	Assess impact of ethnicity on food security, nutrition, sedentary behavior, and BMI	Cross‐sectional study	2‐ to 5‐year‐old children of low‐resource families in Miami, Florida	Risk of reporting bias, lack of generalizability and causal inference
Bender 2014 [22], United States	Reduce sugar beverage intake in a childhood obesity intervention program Vida Saludable	Mixed method	Hispanic mothers with 3‐ to 5‐year‐old children in the United States	Lack of generalizability of specific program adaptations
Christian 2015 [23], Australia	Explore dental service use among migrant children	Cross‐sectional study	Families with 1‐ to 4‐year‐old children from Iraqi, Lebanese and Pakistani backgrounds in metropolitan Melbourne	—
Crespo 2018 [24], United States	Test the efficacy of an obesity intervention to lower BMI in overweight Latino children	Randomized controlled trial	5‐ to 10‐year‐old pediatric patients from a health center in San Diego, California	Target sample size not met, underreported results due to changing inclusion criteria
Cyril 2016 [15], Australia	Explore stakeholders' perspectives on the engagement of CALD communities in childhood obesity initiatives through focus groups	Qualitative research	Healthcare providers of children from CALD groups	Selection bias
Cyril 2017 [14], Australia	Identify barriers and facilitators to the engagement of CALD groups with obesity prevention initiatives	Qualitative research	Vietnamese, Burmese, African, Afghani and Indian parents with a child < 12 years old in one of four disadvantaged areas of Victoria, Australia	Lack of generalizability
Fischer 2014 [25], United States	Examine the role of culture, race, and socioeconomic status in breastfeeding practices	Qualitative research	African American or White pregnant women or mothers of infants < 12 months and self‐reported eligibility/non‐eligibility for the Special Supplemental Nutrition Program for Women, Infants, and Children (WIC)	Lack of individual perspective, recall bias
Frank 2021 [26], United States	Establish a culturally relevant healthy living program and develop the cultural competence of facilitators	Mixed method	Low‐income Latino families with 2‐ to 8‐year‐old children in California	Lack of control group
Furman 2016 [27], United States	Identify characteristics that increase breastfeeding prevalence in a targeted breastfeeding intervention involving community health worker home visits to high‐risk expectant mothers	Non‐randomized experimental study	African American expectant mothers	Lack of randomization, blinding, masking; incomplete adherence to intervention program
Herbst 2019 [28], United States	Culturally adapt management of elevated BMI in young children through the use of motivational interviewing	Quasi‐experimental	Children aged 24 to 66 months in London	Modest sample size, lack of control group
Kaiser 2015 [29], United States	Culturally tailor health education and family night activities intervention of an obesity prevention program	Randomized controlled trial	Families with children aged 2 to 8 years in rural Mexico	—
Leung 2017 [30], United Kingdom	Explore the influence of cultural beliefs on infant feeding practices among immigrant Chinese women	Qualitative research	Chinese mothers who gave birth < 12 months	Modest sample size, selection bias
Little 2013 [31], United States	Implement and evaluate a group prenatal visit program for Japanese women in Michigan	Mixed method	Japanese expectant mothers	Modest sample size, selection bias, unvalidated tools
Lutenbacher 2018 [32], United States	Improve maternal health and child development and increase access to healthcare for pregnant Hispanic women	Randomized controlled trial	Expectant Hispanic women living in metropolitan Tennessee	Lack of control group
Maafs‐Rodriguez 2022 [33], United States	Test the influence of social media in the culturally adapted intervention Kid's Healthy Eating Plate to Spanish‐speaking Latinos	Non‐randomized experimental study	Spanish‐speaking Latino families	Pageviews metrics do not capture all factors influencing a user's engagement with the webpage content
Marshall 2021 [34], Australia	Explore the perspectives of health professionals and Arabic and Chinese mothers on infant feeding supports	Qualitative research	Arabic and Chinese speaking migrant mothers, and health professionals, particularly Child and Family Health Nurses (CFHNs) who deliver routine baby health checks	Analysis and interpretation of data was done in English (different language to when data was collected) which may have affected integrity and credibility of data
Mendoza 2016 [35], United States	Evaluate the Fit 5 Kids program's impact on TV viewing habits of Latino preschoolers	Randomized controlled trial	Latino children aged 3–5 years and their parents	Modest sample size
Nicol 2014 [36], United States	Explore the child oral health knowledge of humanitarian entrant refugees	Qualitative research	Humanitarian entrant refugees and community health nurses in Western Australia	Dental health needs of families were not explored
Pallan 2019 [37], United Kingdom	To culturally adapt an existing children's weight management program towards a South Asian family target population	Qualitative research	Bangladeshi and Pakistani parents and carers of overweight/obese children aged 4–11 years who have been offered the existing children's weight management service	Modest sample size
Ragavan 2018 [38], United States	To examine Asian immigrant parental attitudes and experiences with well‐child visits	Qualitative research	Chinese, Vietnamese, and Asian Indian immigrant parents	Unvalidated tools, culturally sensitive care represented in this study and CCSS survey may not be generalizable to all populations
Ragavan 2020 [39], United States	To explore parental perspectives on the quality and cultural sensitivity of pediatric care	Qualitative research	Participants were parents or caregivers of at least 1 child aged 3–48 months who were accessing the clinic for a well‐child visit for that child	Participants were primarily English‐proficient due to recruitment strategies and not fully representative of target population
Ramachandran 2023 [40], United States	Explore the barriers that pediatricians face in providing care for South Asian children	Qualitative research	Pediatricians who practiced in areas with significant numbers of SA families in New Jersey and New York City, USA	Not reported
Ramos‐Gomez 2014 [41], United States	Provide culturally competent perinatal and infant oral care for underserved, low‐income, and/or minority children and their caregivers	Program evaluation	Underserved, low‐income, and/or minority children aged 0–5 and their caregivers residing in Los Angeles	Not reported
Rehayem 2020 [42], Australia	Explore the experiences, knowledge and influences around infant feeding in Arabic women in Australia	Quantitative descriptive	Arabic background mothers living in Sydney, Australia	Participants were only of Lebanese background and not representative of target population
Rios‐Ellis 2015 [43], United States	To culturally adapt and determine the impact of promotora‐based education on Latina health	Mixed method	Spanish‐speaking expectant Latina women in Los Angeles, California, USA.	Participants lost to follow‐up
Vilasboas 2023 [44], United States	To examine the preferences of Brazilian immigrant parents to develop programs promoting healthful energy balance‐related behaviors	Cross‐sectional study	Brazilian parents with at least one 2‐ to 5‐year‐old child living in Massachusetts, USA	Modest sample size; did not assess all social media platforms
Villalta 2019 [45], United States	Test the effectiveness of a community oral healthcare training program with the goal of reducing childhood caries	Qualitative research	Parents enrolled in Early Head Start (EHS) from Hope Street Margolis Family Center (HSMFC) in Los Angeles, USA	Facilitators were not calibrated and differences between facilitators were not accounted for
Yin 2022 [46], United States	Test a culturally tailored obesity prevention intervention in low‐income, minority preschool age children	Randomized controlled trial	Low‐income, minority preschool age children in Texas, USA.	Lack of blinding, participants lost to follow up may impact the statistical power to detect intervention effect at 8 months post‐test

#### 2.6.2 Quality Appraisal

The Mixed Methods Appraisal Tool (MMAT) [47] was used to assess the quality of studies included due to the wide variety of study types (Table 2). It involved two screening questions and five more criteria questions depending on the study type (qualitative studies, quantitative randomized controlled trials, quantitative non‐randomized studies, quantitative descriptive studies, and mixed‐methods studies). Each question is rated “yes,” “no,” or “cannot tell.” Each included study was critically appraised by two independent assessors (S.W. and Z.C.). Ratings were compared, and conflicts were resolved through discussion. The results of quality appraisals were considered during data analysis but did not result in exclusion of studies in this review.

**TABLE 2:** Quality appraisal against the MMAT [47] criteria.

Author, year	Screening		Qualitative					Quantitative RCT					Quantitative non‐randomized					Quantitative descriptive					Mixed methods					Overall quality score
	S1	S2	1.1	1.2	1.3	1.4	1.5	2.1	2.2	2.3	2.4	2.5	3.1	3.2	3.3	3.4	3.5	4.1	4.2	4.3	4.4	4.5	5.1	5.2	5.3	5.4	5.5	
Asfour 2015	Yes	Yes																Yes	Yes	Yes	No	Yes						***
Bender 2014	Yes	Yes																					Yes	Yes	Cannot tell	Cannot tell	No	*
Christian 2015	Yes	Yes																Yes	Yes	Yes	Yes	Yes						****
Crespo 2018	Yes	Yes						Yes	Yes	Yes	Yes	No																***
Cyril 2016	Yes	Yes	Yes	Yes	Yes	Yes	Yes																					****
Cyril 2017	Yes	Yes	Yes	Yes	Yes	Yes	Yes																					****
Fischer 2014	Yes	No	Yes	No	Yes	Yes	Cannot tell																					*
Frank 2021	Yes	Yes																					Yes	Yes	Yes	Cannot tell	Yes	***
Furman 2016	Yes	Yes											Yes	Yes	No	No	Yes											**
Herbst 2019	Yes	Yes																Yes	No	Yes	Yes	Yes						***
Kaiser 2015	No	Cannot tell						Yes	Cannot tell	Cannot tell	No	No																U
Leung 2017	Yes	Yes	Yes	Cannot tell	Yes	Yes	Yes																					***
Little 2013	Yes	Yes																					Yes	Yes	Yes	Yes	Cannot tell	***
Lutenbacher 2018	Yes	Yes						Yes	Yes	Yes	Yes	Yes																****
Maafs‐Rodriguez 2022	Yes	Yes											Yes	Yes	Yes	Cannot tell	Yes											***
Marshall 2021	Yes	Yes	Yes	Yes	Cannot tell	Yes	Yes																					***
Mendoza 2016	Yes	Yes						Yes	Yes	Yes	No	Yes																***
Nicol 2014	Yes	Yes	Yes	Yes	Yes	Yes	Yes																					****
Pallan 2019	Yes	Yes	Yes	Yes	Yes	Yes	Yes																					****
Ragavan 2018	Yes	Yes	Yes	Yes	Yes	No	Yes																					***
Ragavan 2020	Yes	Yes	Yes	Yes	Yes	Yes	Yes																					****
Ramachandran 2023	Yes	Yes	Yes	Yes	Yes	Yes	Yes																					****
Ramos‐Gomez 2014	No	Cannot tell																Yes	Cannot tell	Cannot tell	Cannot tell	Cannot tell						U
Rehayem 2020	Yes	No	Yes	No	Yes	Yes	Yes																					*
Rios‐Ellis 2015	Yes	Yes																					Yes	Yes	Yes	Yes	No	***
Vilasboas 2023	Yes	Yes																Yes	Yes	Yes	Yes	Yes						****
Villalta 2019	Yes	Yes											Yes	Yes	Yes	Yes	Yes											****
Yin 2022	Yes	Yes						Yes	Yes	Yes	No	Yes																***

*Note:* * meets 25% of the MMAT criteria. ** meets 50% of the MMAT criteria. *** meets 75% of the MMAT criteria. **** meets 100% of the MMAT criteria.

Abbreviation: U, unclassified.

#### 2.6.3 Data Synthesis

Included studies were categorized into 5 distinct groups according to the ways cultural competence was explored. These categories included studies that (i) assessed cultural competence, (ii) were cultural adaptations of interventions, (iii) provided perspectives of the CALD groups on preventive healthcare services, (iv) provided perspectives of healthcare workers, and (v) involved healthcare cultural competency training. Studies were then grouped by the health behaviors explored, the recipients of the study, the cultural groups explored, key characteristics associated with cultural competence and responsiveness, and finally key outcomes associated with cultural competence and responsiveness. The search was reported according to Preferred Reporting Items for Systematic Reviews and Meta‐Analyses (PRISMA) [48] guidelines. All results were presented in tables with the exception of results identifying barriers and facilitators to CALD healthcare access.

## 3 Results

### 3.1 Study Selection

**FIGURE 1:**
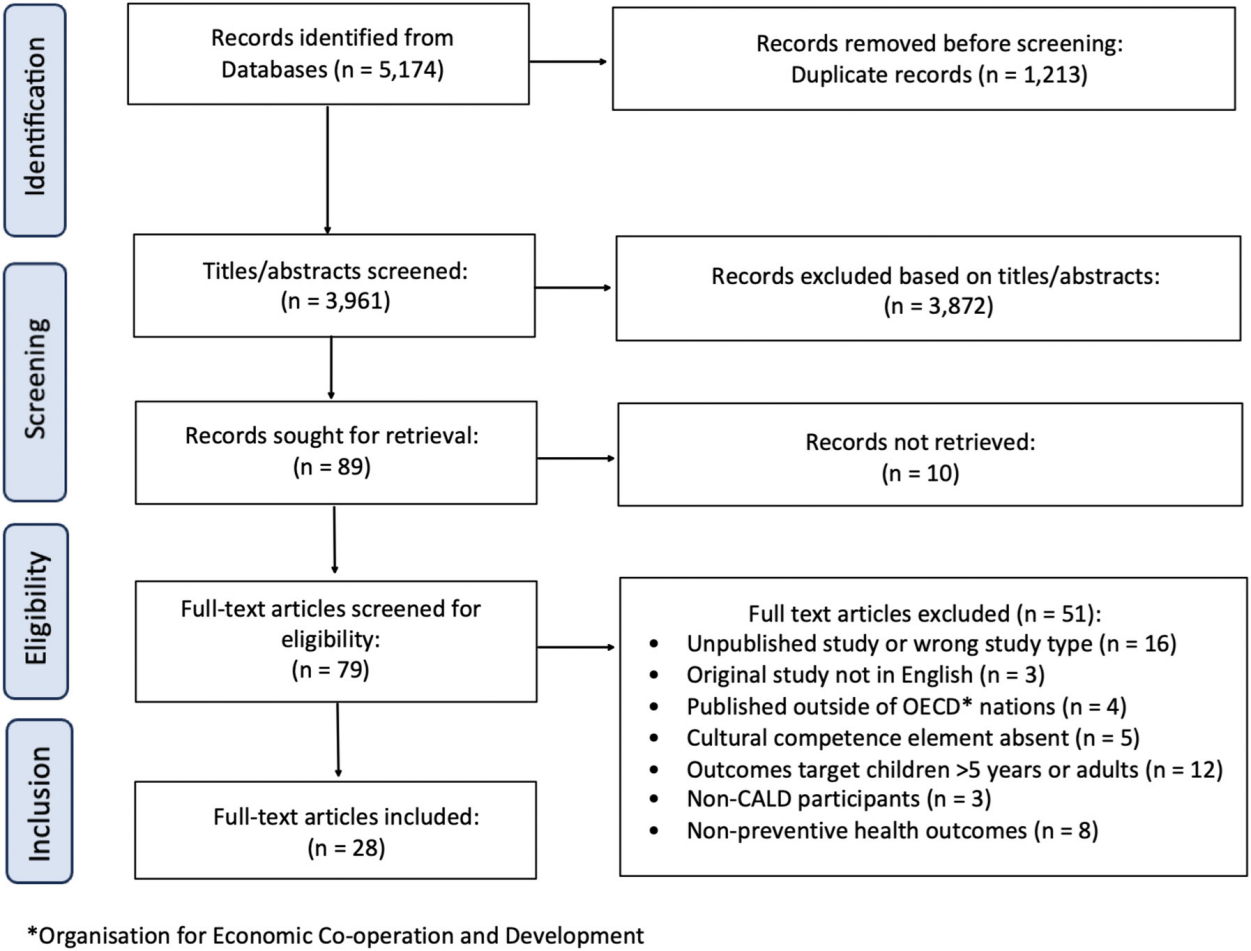
PRISMA‐ScR [48] diagram of search findings and study selection.

The searches yielded 5174 records, with 1213 duplicates removed, leaving 3961 records that were screened by title and abstract by two independent reviewers. A total of 89 records were sought for retrieval of full texts. Since the full text of 10 records could not be found, 79 full‐text articles were evaluated by two independent reviewers (S.W. and Z.C.) against the eligibility criteria. Finally, a total of 28 articles [14, 15, 21, 22, 23, 24, 25, 26, 27, 28, 29, 30, 31, 32, 33, 34, 35, 36, 37, 38, 39, 40, 41, 42, 43, 44, 45, 46] were included in this review (Figure 1).

### 3.2 Description of Studies

Descriptions of included studies are reported in Table 1 including aims, type of study, target group and setting, a brief description of the cultural competence process, and outcomes measured. Studies mainly aimed to improve maternal and child health behaviors (*n* = 7), explore perspectives of CALD groups and their service providers on preventive health (*n* = 7), prevent childhood obesity (*n* = 4), and promote preventive health education (*n* = 4). The majority of studies were published in the United States of America (*n* = 21), with the remaining from Australia (*n* = 5) and the United Kingdom (*n* = 2). Most studies were qualitative (*n* = 11) or randomized control trials (*n* = 5), with a large variety of other study types including cross‐sectional (*n* = 3), mixed method (*n* = 4), non‐experimental observational (*n* = 1), non‐randomized experimental (*n* = 2), quantitative descriptive (*n* = 1), and quasi‐experimental (*n* = 1). Cultural groups explored include Black (*n* = 8), East Asian (*n* = 6), Hispanic/Latino (*n* = 14), Middle Eastern (*n* = 7), South Asian (*n* = 4), Vietnamese (*n* = 2), and unknown (*n* = 2; specific cultural groups were not reported).

#### 3.2.1 Exploration of Culturally Competent and Responsive Care Within Studies

The key strategies and characteristics associated with culturally competent and responsive care employed by studies are listed in Table 3. Strategies were identified as the primary methods that explored cultural competency. The key strategies were cultural adaptations to an intervention (*n* = 10), exploring perspectives of CALD groups (*n* = 8), healthcare cultural competence training (*n* = 5), assessment of cultural competence (*n* = 3), and exploring perspectives of healthcare professionals (*n* = 2). Characteristics were identified as the most common characteristics between study methods that explored cultural competency, for example, a process or tool. The key characteristics common among the studies included the use of bilingual facilitators (*n* = 12), distribution of educational resources (*n* = 8), language‐modified materials or program content (*n* = 8), use of focus groups (*n* = 7), culturally tailored content (*n* = 6), use of research to inform the study (*n* = 6), targeting parenting practices (*n* = 3), and motivational interviewing (*n* = 2). Four [26, 28, 32, 43] out of five studies investigating healthcare training involved bilingual facilitators, whereas the fifth [41] involved specific cultural, linguistic, and health literacy training. Focus groups were used commonly, which enabled deeper exploration of shared and individual perspectives [15] in studies exploring the perspectives of CALD groups, although one study utilized the nominal group technique instead [14].

**TABLE 3:** Strategies and characteristics associated with culturally competent and responsive care (*n* = 28).

Intervention characteristics	Description	*n*	References
Strategies			
Cultural adaptations of interventions	Interventions that were modified to suit the participant's language, culture, and context	10	[21, 22, 24, 27, 29, 31, 33, 35, 37, 46]
Assessment of cultural competence	Studies that assessed the cultural competence of service providers, for example, clinical assessment and patient assessment	3	[38, 39, 45]
Perspectives of CALD groups	Studies that explored the perspectives of CALD groups receiving childhood preventive care	8	[14, 23, 25, 30, 34, 36, 42, 44]
Perspectives of healthcare professionals	Studies that explored the perspectives of healthcare professionals providing care to CALD groups	2	[15, 40]
Healthcare cultural competence training	Interventions that provided training to service providers to develop cultural competency	5	[26, 28, 32, 41, 43]
Characteristics			
Bilingual facilitator	Study was facilitated by bilingual staff who interacted with participants in their own preferred language	12	[22, 24, 26, 29, 32, 34, 37, 38, 43, 45, 46]
Educational resources	Educational materials were distributed to participants to increase health knowledge	8	[26, 28, 30, 33, 37, 43, 44, 46]
Focus groups	Focus groups were used to deliver the program	7	[22, 25, 26, 29, 34, 36, 42]
Culturally tailored content	Content of study were tailored to the culture of participants, for example, culture‐specific food, images, and practices	6	[22, 26, 31, 33, 35, 36]
Home intervention	Intervention based at the participant's home	3	[27, 32, 46]
Motivational interviewing	A counseling technique that seeks to increase the participant's motivation for change	2	[26, 28]
Formative research methods to inform study design	Results of qualitative research or literature review were used to inform adaptations or design of the study	6	[22, 24, 26, 29, 35, 37]
Targeted parenting practices	For example, sleep and nutrition	3	[24, 37, 38]
Language‐modified materials	Materials used in the study were translated into the participant's preferred language, for example, surveys and information booklets	8	[14, 21, 22, 26, 31, 33, 34, 35]

Table 4 details the key processes and ways that cultural competence was applied to included studies. Nine studies utilized frameworks, models, or theories to explore cultural competency, where most studies built upon the *Promotores* model (*n* = 5). The target health behaviors and significant outcomes are also listed, where nutrition was the most common target behavior (*n* = 16), followed by physical activity (*n* = 7), preventive care (*n* = 7), sedentary behavior (*n* = 5), and oral health (*n* = 4).

**TABLE 4:** Exploration of cultural competency in included studies (*n* = 28).

First author, year, reference, country	Target cultural group	Intervention name	Cultural adaptation theory, model or framework	Brief description of application of cultural competence	Cultural competence strategy	Target health behavior	Significant outcomes
Asfour 2015 [21], United States	Hispanic and Latino families	Healthy Caregivers‐Healthy Children (HC2)	—	Cultural adaptations were made to a weight management program; translation of program materials	Cultural adaptations to an intervention	Nutrition	Significant relationship between food security and child consumption of fruit/vegetables, child consumption of unhealthy foods, and sedentary behavior. Haitians report greater consumption of fruit/vegetables, sedentary behavior, and consumption of unhealthy foods with food security, whereas Cubans report less consumption of unhealthy foods
Bender 2014 [22], United States	Hispanic mothers with children	Vida Saludable	Resnicow et al.'s [49] surface and deep adaptations; Kreuter et al.'s [50 ] five categorical strategies for adaptation and concepts of targeting and tailoring; and Eremenco et al.'s [51] translation guidelines for programmaterials; *Promotores de Salud* model [52]	Cultural adaptations to Vida Saludable to target a Hispanic population; bilingual facilitators, promotora‐led; translation of program materials; culturally tailored content (pictures, beverages, and cultural views)	Cultural adaptations to an intervention	Nutrition, physical activity	—
Christian 2015 [23], Australia	Iraqi, Lebanese, and Pakistani immigrant families	—	—	Perspectives of CALD groups; bilingual facilitators; identified barriers; identified facilitators	Explores cultural issues/barriers to healthcare services	Oral health	88% of migrant children had never visited the dentist. Parents reported “no reason to visit the dentist” the most. Out of those parents, 22% of children experienced dental caries
Crespo 2018 [24], United States	Latino pediatric patients	The Luces intervention	Socioecological Model for Latino Health Promotion [53]	Cultural adaptations to an intervention Luces; bilingual facilitators; guided by the Socioecological Model for Latino Health Promotion	Cultural adaptations to an intervention	Nutrition, physical activity, sedentary behavior	Total and trunk per cent fat was significantly lower in intervention children versus control group
Cyril 2016 [15], Australia	CALD communities	—	—	Perspectives of CALD groups; addressed barriers and facilitators; focus groups; identified barriers and facilitators	Perspectives of healthcare providers	Preventive care	—
Cyril 2017 [14], Australia	Disadvantaged Vietnamese, Burmese, African, Afghani, and Indian families	—	—	Perspectives of CALD groups; bilingual facilitators; identified barriers and facilitators; translation of survey materials	Perspectives of CALD population; Explores cultural issues/barriers to health services	Preventive care	—
Fischer 2014 [25], United States	African American mothers and expectant mothers	—	—	Perspectives of CALD groups; focus groups; identified barriers and facilitators	Perspectives of CALD population; explores cultural issues/barriers to health services	Nutrition	—
Frank 2021 [26], United States	Low‐income Latino families	—	*Promotores de Salud* model [52]	Healthcare cultural competence training; bilingual facilitators; promotora‐led; motivational interviewing; qualitative research to inform study	Assessment of cultural competence; healthcare cultural competence training	Nutrition	Increased service provider knowledge about influences on and skills for addressing Latino health; increased provider confidence in delivering CALD obesity interventions and curriculums; increased child fruit and vegetable intake; decreased sugar beverage consumption
Furman 2016 [27], United States	African American expectant mothers	—	—	Cultural adaptation of an intervention; home intervention	Cultural adaptations to an intervention	Nutrition	Odds of any breastfeeding were increased with participation in curricular modules and a postpartum visit; odds of exclusive breastfeeding was significantly increased by a postpartum visit
Herbst 2019 [28], United States	Asian, Black, and Hispanic/Latino children	—	—	Cultural adaptations to an intervention; addressed barriers; motivational interviewing; information handouts	Cultural adaptations to an intervention	Preventive care	Provider documentation of weight and development of healthy habit goals during well‐child visits improved post‐intervention; families were more likely to return for visits post‐intervention
Kaiser 2015 [29], United States	Hispanic families in rural Mexico	Niños Sanos, Familia Sana (Healthy Children, Healthy Family)	*Promotores de Salud* model [52]	Cultural adaptations to an intervention; bilingual facilitators focus groups; promotora‐led; qualitative research to inform study	Cultural adaptations to an intervention	Nutrition, physical activity	—
Leung 2017 [30], United Kingdom	Immigrant Chinese mothers	—	—	Perspectives of CALD groups; identified barriers and facilitators	Perspectives of CALD population; explores cultural issues/barriers to health services	Nutrition	—
Little 2013 [31], United States	Japanese expectant mothers	—	—	Cultural adaptations made to a prenatal group visit program; bilingual facilitators; translation of survey materials; culturally‐tailored content	Cultural adaptations to an intervention	Preventive care	Most participants reported educational topics were “covered” or “covered well” in the culturally adapted prenatal visit group. Participants reported improved understanding about prenatal care and preparation for newborn care
Lutenbacher 2018 [32], United States	Hispanic expectant mothers	The Maternal Infant Health Outreach Worker (MIHOW) program	—	Healthcare cultural competence training; bilingual facilitators; home intervention	Healthcare cultural competence training	Nutrition	Intervention group had a significant increase on breastfeeding self‐efficacy and exclusivity, safe sleep practices, and infant stimulation
Maafs‐Rodriguez 2022 [33], United States	Spanish‐speaking Latino families	Kid's Healthy Eating Plate (HEP)	Framework for Reporting Adaptations and Modifications‐Expanded (FRAME) [54]	Cultural adaptations to an intervention, the Kid's Healthy Eating Plate (HEP); culturally‐tailored content (food); translation of materials; social media	Cultural adaptations to an intervention	Nutrition	—
Marshall 2021 [34], Australia	Arabic‐ and Chinese‐speaking migrant mothers	Healthy Beginnings program	—	Perspectives of CALD groups (Arabic‐ and Chinese‐speaking migrant mothers); bilingual facilitators; focus groups; translation of materials	Perspectives of CALD population; Perspectives of healthcare providers	Nutrition, physical activity, sedentary behavior	—
Mendoza 2016 [35], United States	Latino children	Fit 5 Kids	—	Cultural adaptation of the Fit 5 Kids intervention aimed at Latino families; culturally tailored content (words, phrases, songs, and poetry); qualitative research to inform study; translation of materials	Cultural adaptations to an intervention	Sedentary behavior	Decrease in TV viewing > 25 min daily post‐intervention
Nicol 2014 [36], United States	South‐East Asian, Middle Eastern, and African refugees	—	—	Perspectives of CALD groups; focus groups; identified barriers and facilitators	Perspectives of CALD population; opportunities for Change model; Perspectives of healthcare providers; explores cultural issues/barriers to health services	Oral health	—
Pallan 2019 [37], United Kingdom	Bangladeshi and Pakistani families	First steps (adapted)	Framework for design and evaluation of complex health interventions [55]	Cultural adaptations made to weight management program ‘First Steps’; qualitative research to inform study; guided by theoretical frameworks for the development of behavior change interventions and adaption typology; identified barriers and facilitators	Cultural adaptations to an intervention	Nutrition, physical activity	—
Ragavan 2018 [38], United States	Chinese, Vietnamese, and Indian immigrant parents	—	—	Assessment of cultural competence; bilingual facilitators; translation of materials English	Assessment of cultural competence; perspectives of CALD population	Preventive care	Parents born overseas versus in the United States reported significantly higher culturally sensitive care scores; Haitian parents reported significantly lower culturally sensitive care scores compared to non‐Hispanic parents; significant association between parent‐reported culturally sensitive care and increased well‐child visit quality
Ragavan 2020 [39], United States	Black (African American or African), Asian, Hispanic/Latino, and Haitian parents or caregivers	—	Brotanek et al.'s [56] framework	Language‐modified materials (surveys); parent‐reported surveys; culturally sensitive care was measured through the Clinicians Cultural Sensitivity Survey (CCSS), which was modified using the Brotanek et al. framework; well‐child visit quality was measured through the Promoting Healthy Development Survey (PHDS); identified barriers to culturally sensitive care	Assessment of cultural competence; perspectives of CALD population	Preventive care	—
Ramachandran 2023 [40], United States	South Asian children	—	—	Perspectives of healthcare professionals; identified barriers.	Perspectives of healthcare providers	Preventive care	—
Ramos‐Gomez 2014 [41], United States	Disadvantaged or minority families	Infant Oral Care Program (IOCP)	—	Healthcare cultural competence training provided to community partners; culturally‐tailored content (cultural and country norms)	Healthcare cultural competence training	Oral health	—
Rehayem 2020 [42], Australia	Arabic background mothers (predominantly Lebanese)	—	—	Perspectives of CALD groups (Arabic women); focus groups	Perspectives of CALD population	Nutrition	—
Rios‐Ellis 2015 [43], United States	Spanish‐speaking expectant Latina mothers	Comienzo Saludable, Familia Sana (Healthy Start, Healthy Family)	*Promotores de Salud* model [52]	Cultural adaptation of an intervention the Salud con Hyland's Project: Comienzo Saludable, Familia Sana; bilingual facilitators; promotora‐led; educational resources (calendar to track infant feeding and development); charlas	Healthcare cultural competence training; other: Community‐based version of a previous experimentally designed culturally tailored intervention	Nutrition	Significant increase in intention to breastfeed exclusively after the first *charla* in home and mixed location groups; significant increase in proportion of women planning to breastfeed for longer durations for women who received two *charlas* at home; significant change in planned age to introduce solids to later ages; increase in knowledge regarding foods not to introduce in first year of life for all groups
Vilasboas 2023 [44], United States	Brazilian immigrant parents	—	—	Perspectives of Brazilian immigrant parents are explored in this study. Preferences for content, intervention modality and language of an intervention to promote health‐related behaviors are explored using the family ecological model (FEM) as a guide	Perspectives of CALD population	Nutrition, physical activity, sedentary behavior	Higher proportion of mothers versus fathers report being interested in learning about most EBRBs. Higher proportion of fathers versus mothers report being interested in reducing child consumption sugary beverages and increasing water consumption. Parents were least interested to learn about promoting water consumption and receiving adequate sleep
Villalta 2019 [45], United States	Latino parents	Early Head Start	*Promotores de Salud* model [52]	Healthcare cultural competence training; bilingual facilitators	Assessment of cultural competence; healthcare cultural competence training	Oral health	Significant increase in service provider's knowledge for age that a child can brush their teeth independently, age to use fluoridated toothpaste, tooth decay prevention, and timing child's first dental visit
Yin 2022 [46], United States	Latino/Hispanic children	Miranos	—	Cultural adaptations to an obesity prevention program targeted at Latino children; addressed barriers and facilitators; bilingual facilitators; culturally tailored content (images, food); home intervention	Cultural adaptations to an intervention	Nutrition, physical activity, sedentary behavior	BMI z‐scores and weight‐for‐age z scores were reduced in the center and home‐based intervention group compared to the control group

Outcomes greatly varied, with some studies reporting qualitative or non‐significant outcomes. Significant outcomes identified included improved child nutrition (*n* = 4), increased engagement with health services (*n* = 3), and increased health knowledge (*n* = 2). Notably, studies exploring healthcare cultural competence training all resulted in significant outcomes. Despite the sizeable number of included studies, only four were delivered as randomized control trials, with the majority conducted as qualitative research into the experiences of CALD families and practitioners. Furthermore, all five lower quality studies [22, 25, 29, 41, 42] (determined as meeting less than or equal to 25% of the MMAT criteria or unclassified) did not report significant findings, although one [25] was qualitative in design. Meanwhile, all other studies either reported qualitative data or significant outcomes, except for a non‐randomized experimental study [33].

#### 3.2.2 Barriers and Facilitators to Culturally Competent Care

Although there were common characteristics and outcomes that were associated with culturally competent care, many barriers and suggestions for improvements were identified throughout the studies. Barriers to children of CALD backgrounds receiving appropriate preventive healthcare included:

- Accessibility and utilization, for example, cost, language, transport, and waiting lists [23, 36];
- Parental influence, for example, lack of health literacy, different health practices, and aversion to healthcare due to previous experiences [14, 36];
- Providers' lack of language and cultural understanding [23, 30, 38]; and
- Competing priorities in the post‐immigration resettlement phase [36].

Identified areas of improvement and facilitators to culturally competent care included considering:

- Involving bilingual and bicultural facilitators and leaders [14, 38];
- Culturally tailoring content, for example, measurement charts customized to race/ethnicity, and knowledge of how illnesses are conceptualized outside of OECD nations [38];
- Engaging family in visits or interventions [34, 39, 40];
- Engaging in ethnic and cultural community groups [14];
- Increased accessibility to targeted community programs [23, 34, 44];
- Increased provider knowledge in cultural beliefs and traditions, particularly complementary and alternative medicines [30, 38, 39, 40, 42];
- Increased social awareness and acceptability of different parenting practices, particularly in sleep and child nutrition [25, 38, 42]; and
- Mentoring and goal setting [25].

### 3.3 Quality Appraisal

Only 10 out of 28 studies met 100% of the MMAT [47] criteria. Five studies were determined to be meeting less than or equal to 25% of the criteria or unclassified. Quality appraisal did not lead to the exclusion of any studies.

## 4 Discussion

This scoping review found 28 studies that explored cultural competency within the area of childhood preventive health, most of which were published within the United States (*n* = 21). A majority of studies aimed to improve maternal and child health behaviors (*n* = 7) and involved cultural adaptations of interventions (*n* = 10), commonly utilizing bilingual facilitators and language‐modified content or materials. Other studies explored the perspectives of CALD families or health professionals delivering services to CALD families, majority of which were qualitative studies. Some studies assessed cultural competency in health services delivered to CALD families or delivered cultural competence training to health professionals. Few studies utilized a theory, model, or framework to explore cultural competency, but of those that did, the *Promotores de Salud* model [49] was most common (*n* = 5). This review investigated a range of studies involving cultural competency, but not necessarily assessing the effects of cultural competency on CALD obesity prevention. Of the studies that reported significant results, most involved bilingual health educators, including nurses and community healthcare workers, who provided health education, support, and resources. These successful interventions engaged with parents or families in their first language, and often staff were trained thoroughly for program delivery. Interventions were commonly delivered as home visits; however, some involved workshops in the community, and would occur consistently over a few months, usually spanning over a few hours per week. A portion of studies reported nonsignificant findings, of which most were critically appraised as low quality via the MMAT criteria, or reported qualitative data. Hence, further robust study, in particular randomized controlled trials, testing the effectiveness of cultural competence strategies should be a focus in future research.

When examining the ways cultural competence was explored within studies, cultural adaptations of interventions were the most common category. Notably, all studies that involved healthcare cultural competence training resulted in significant findings in a key outcome associated with culturally competent and responsive care. This suggests that cultural training provided to health professionals has an impact on outcomes of children of CALD backgrounds, and should be an area of focus in future research and practice as well. Among the cultural competency strategies utilized, characteristics found to be common included the utilization of bilingual facilitators (e.g., program staff) and translation of materials into the participant's preferred language (e.g., program materials, handouts, and surveys). Four of these studies involved interventions that were led by a promotora—a Latino/Hispanic community member that is specially trained in providing health education. Use of community health workers such as promotoras is an inexpensive and resourceful strategy in effectively addressing Latino health disparities [43]. The use of culturally tailored content, such as using culturally relevant images or food, was also commonly seen in studies. Hence, the development of diverse linguistic and cultural training to provide culturally tailored health services can have a large impact on providing better care to this population. Additionally, educational resources related to health promotion were identified as a common characteristic. Greater production and distribution of culturally and linguistically appropriate educational resources could address barriers such as a low health literacy.

Studies greatly varied in target population, methods, outcomes, and in the ways cultural competence was explored. Jongen et al.'s [17] scoping review of cultural competency in the healthcare workforce found inconsistent definitions of cultural competency and varied cultural differences across cultural groups in 64 identified studies. They concluded that approaches to cultural competence are distinct and different to each cultural group, which is reflected in the variability in the results of our review. Moreover, Jongen et al. [17] similarly identified cultural competency training as a common strategy utilized by health services to improve health services delivered to CALD groups. Identified approaches to training reported include (i) developing broad knowledge and skills applicable to multicultural groups and (ii) learning specific beliefs and behaviors of individual cultures. Notably, their review also considered discrimination and racism as important issues to address in cultural competency training which our review did not examine, in particular issues surrounding practitioner bias and how it affects practitioner care. However, their results indicated only one intervention that addressed the issue of stereotyping within the cultural competency training program [57]. More research should seek to explore whether addressing racism and discrimination in cultural competency training improves the care delivered to CALD populations.

Culturally competent strategies have been shown to improve health outcomes in other health areas. Notably, Hasnain et al.'s [58] systematic review demonstrated that culturally competent interventions are effective in improving the health outcomes of patients who are CALD with disabilities. Hasnain et al. [58] identified strategies that ensured interventions were culturally relevant, all of which were comparable to the cultural competency strategies and characteristics identified in our review. These include running programs in the participants' preferred language and accommodating for communication barriers (translated materials and interpreters), addressing inadequacies in health education and behavior (health promotion), and incorporating culturally‐relevant elements into interventions and tools used. One particular cultural competent initiative suggested by Hasnain et al. [58] that our study did not identify was the use of racial and ethnic concordance between participants and health professionals. Similarly, Clifford et al.'s [59] systematic review exploring cultural competency in Indigenous populations identified the importance of employing an Indigenous workforce as a cultural competency strategy. Although our review identified the use of bilingual and bicultural facilitators of programs was a common characteristic across studies, Hasnain et al. [58] and Clifford et al.'s [52] studies implied that just cultural competency training is not enough to improve health outcomes in Indigenous and CALD populations. Instead, greater diversity and inclusion of Indigenous and CALD groups within the healthcare workforce will greatly benefit these populations.

A range of outcomes were described throughout studies, including increased engagement with healthcare services, child health behaviors, child weight, and parental practices (in particular infant feeding). Out of 28 included studies, most studies explored nutritional outcomes (*n* = 16). Few studies produced significant outcomes, but these included increased engagement with healthcare services and increased health literacy, where notable improvement of breastfeeding practices (*n* = 4) was the most common. Significant behavioral outcomes of note include improved nutritional intake in Frank et al.'s [26] study and decreased sedentary behavior in the Fit 5 Kid randomized control trial [35]. There were no significant changes to child BMI, although Crespo et al.'s [24] study noted a reduction in percent body fat post‐intervention and Yin et al.'s [46] study reported lower BMI z‐scores and weight‐for‐age z‐scores in the intervention group. Further studies addressing the effectiveness of culturally competent strategies on childhood behavioral and weight outcomes should be a focus in future research.

Many barriers and facilitators were identified throughout studies that explored the perspectives of CALD groups and health professionals. Efforts should be made to adapt interventions such that they involve bilingual and bicultural facilitators, culturally tailor content, and engage patient families in visits. Providers should further develop cultural competency through increasing knowledge in cultural beliefs and traditions, learning about contemporary medicine, and developing further social awareness regarding different cultural parenting practices. Furthermore, addressing CALD accessibility to health services is crucial. Many studies identified waiting times, language barriers, cost, and other priorities after resettling post‐immigration as barriers to accessing healthcare. Further funding and development of targeted community outreach programs can alleviate some of these issues. Engaging CALD groups in ethnic and cultural community groups has also been noted as a prominent facilitator to culturally competent care. Assessing the strengths and limitations of studies explored in this review, many qualitative studies involving focus groups or interviewing reported social desirability and recall bias. Furthermore, most studies that culturally adapted interventions reported limited generalizability of results beyond the target population and a limited sample size. As many of the target populations were specific to ethnicity, age, and setting, this greatly limits the application of cultural competency strategies described in studies on the CALD population as a whole. As such, our review identifies a need for a greater range of studies exploring cultural competency in a greater diversity of CALD populations.

### 4.1 Strengths and Limitations

Our review is one of the first to explore cultural competence and responsiveness of health services within preventive and childhood health research. Strengths of our review included the comprehensive review process following the JBI methodology and PRISMA‐ScR [48] guidelines across five databases, use of two or more independent reviewers during the review process, and prospective pre‐registration of the review on Open Science Framework. The main limitation of our study was that it did not cover all relevant literature within these areas of research, as it was limited to peer‐reviewed literature published in OECD [18] countries, with gray literature excluded. Due to time constraints, hand referencing was not performed, and studies where the full text could not be found were excluded. Furthermore, changes were made to our eligibility criteria in regard to the types of sources included and age limit of the target population up until the full‐text screening stage, which may have led to exclusion of relevant studies. Inclusion of literature addressing Indigenous populations within our eligibility criteria would greatly benefit cultural competence of preventive health services, particularly in countries such as Australia.

Notably, the term “cultural competence” has been criticized over recent years for its limitations in restricting culture to a single static view in which providers adopt a single end “goal” to master. This approach subsequently reinforced cultural stereotypes, dismissed intersectionality, and encouraged power imbalances between patients and providers [60, 61]. Meanwhile, new terminology such as “cultural humility,” first introduced by Tervalon and Murray‐Garcia in 1998 [62], has been increasingly adopted in literature. Cultural humility is defined as a lifelong process of self‐reflection and acknowledgement of one's biases and limitations in order to deepen understanding of other cultures. This review acknowledges the paradigm shift towards terms such as cultural humility, which aims to cultivate patient‐centered care and account for the many complexities of culture [63].

### 4.2 Implications for Future Practice

The key implications from this review are to identify areas of research and practice that have the potential to improve healthcare services delivered to families and children of CALD background. These include (i) interventions delivered by culturally and linguistically competent facilitators, (ii) continued healthcare training in cultural competence and responsiveness, (iii) studies that aim to explore and improve the health behaviors of sleep and oral health in CALD communities, and (iv) studies that aim to address barriers to healthcare that have been repeatedly experienced by CALD communities. Such future research, with ours, can continue to inform preventive health initiatives directed at CALD communities at both practice and policy levels. Although CALD communities may face adversity within healthcare, the recognition of the need for culturally tailored and sensitive care by frontline clinicians can change this. Patient‐centered care has been increasingly recognized as important, and practicing this with patients from CALD backgrounds may involve a deeper understanding of the barriers and inequities that they face, as well as strategies to overcome these. Clinicians should consider integrating the greater use of bicultural and bilingual facilitators, translated materials, and culturally tailored content into the delivery of healthcare. Furthermore, strengthening guidelines around childhood obesity prevention and establishing cultural competence training in healthcare may improve access to healthcare and the health inequities of CALD communities.

## 5 Conclusion

This review highlights that preventive health services delivered to CALD families are still an area of research and practice that is lacking cultural competence and responsiveness. Efforts should be made to increase research in services that improve sleep and oral health of children of CALD background. Key characteristics and outcomes associated with cultural competence and responsiveness in this population—such as use of bilingual facilitators, focus groups, and language‐modified resources—were identified. A multidisciplinary and community‐centered approach towards addressing health issues at a provider, community, and policy‐making level should be utilized to improve future practice and ultimately reduce health disparities within the CALD community.

## Conflicts of Interest

The authors declare no conflicts of interest.

## Appendix A Search Strategy Developed for Medline via Ovid

**Table:**

1	Exp Infant/or child/or child health services/
2	(infan* or child* or pre?school or p?ediatric or toddler or baby or babies).mp.
3	1 or 2
4	Exp Cultural Diversity/or Cultural Competency/or Culturally Competent Care/
5	((Cultur* or ethnic* or (multi?cultural or multi cultural) or (cross cultur* or cross?cultur*)) adj4 (competen* or modif* or sensitiv* or specific* or focus* or relevan* or innovat* or tailor* or adapt*)).mp.
6	4 or 5
7	Exp sleep/
8	(sleep and (quality or duration or time or latency or stages or onset)).mp.
9	Exp physical activity/or exercise/or motor activity/
10	((physical and inactivity) or (physical and activity) or (tummy and time) or supine or (movement and skills) or (physical and education) or sedentary or sport or danc*).mp.
11	Exp diet/or nutrition/or feeding behavior/
12	(diet* or nutrition* or health* adj2eat* or “Feeding behavio?r” or respons* feed* or breastfe* or (infant adj2 feed*)).mp.
13	exp Oral Health/or exp Dental Care/
14	((Oral and (care or hygiene or health)) or (dental and (care or hygiene or health)) or dental service).mp.
15	exp Health promotion/or Health education/or Preventive health/
16	or/7–15
17	3 and 6 and 16
18	Limit 17 to (English language and year = “2013–current”)

## Appendix B Data Extraction Table

**Table:**

Title of study	
Year of publication	
Origin	
Type of study	
Aim of study	
Study setting	
Study population	
Methodology	
How is cultural competence explored?	
Description of cultural competence element/process	
Rationale for cultural competence element/process	
To what extent was the target population involved?	
Who led/undertook the cultural competence element/process?	
Outcome	
Most relevant findings	
Comments	
